# Electron tomography of rabbit cardiomyocyte three-dimensional ultrastructure

**DOI:** 10.1016/j.pbiomolbio.2016.05.005

**Published:** 2016-07

**Authors:** Eva A. Rog-Zielinska, Callum M. Johnston, Eileen T. O’Toole, Mary Morphew, Andreas Hoenger, Peter Kohl

**Affiliations:** a National Heart and Lung Institute, Imperial College London, UK; b Department of Molecular, Cellular and Developmental Biology, University of Colorado, Boulder, CO, USA; c Institute for Experimental Cardiovascular Medicine, University Heart Centre Freiburg – Bad Krozingen, Medical School of the University of Freiburg, Germany

**Keywords:** T-tubules, Sarcoplasmic reticulum, Microtubule, 3D imaging, ER, endo-plasmic reticulum, ET, electron tomography, FIB, focused ion beam, Mito, mitochondria, MT, microtubules, SBF, serial block face, SEM, scanning electron microscopy, SL, sarcomere length, (j)SR, (junctional) sarcoplasmic reticulum, (n)SR, (network) sarcoplasmic reticulum, TEM, transmission electron microscopy, T-tub, T-tubules, 2D, two-dimensional, 3D, three-dimensional

## Abstract

The field of cardiovascular research has benefitted from rapid developments in imaging technology over the last few decades. Accordingly, an ever growing number of large, multidimensional data sets have begun to appear, often challenging existing pre-conceptions about structure and function of biological systems. For tissue and cell structure imaging, the move from 2D section-based microscopy to true 3D data collection has been a major driver of new insight. In the sub-cellular domain, electron tomography is a powerful technique for exploration of cellular structures in 3D with unparalleled fidelity at nanometer resolution.

Electron tomography is particularly advantageous for studying highly compartmentalised cells such as cardiomyocytes, where elaborate sub-cellular structures play crucial roles in electrophysiology and mechanics. Although the anatomy of specific ultra-structures, such as dyadic couplons, has been extensively explored using 2D electron microscopy of thin sections, we still lack accurate, quantitative knowledge of true individual shape, volume and surface area of sub-cellular domains, as well as their 3D spatial interrelations; let alone of how these are reshaped during the cycle of contraction and relaxation. Here we discuss and illustrate the utility of ET for identification, visualisation, and analysis of 3D cardiomyocyte ultrastructures such as the T-tubular system, sarcoplasmic reticulum, mitochondria and microtubules.

## 1 Introduction

The predominantly reductionist scientific approach, whilst offering important insight that otherwise might be obscured, is not without limitations (Asano, et al., 2016), as even the most detailed knowledge of individual components alone is often insufficient to predict the behaviour of complex biological systems. However, despite the fact that no biological system exists in isolation, we often approach and study biological structures as if they were independent of their environment.

A cell is one example of a complex system whose individual components interact with one another, and with their environment, in time and space. These interactions influence function and structure of each individual component. Cellular processes are performed by molecular assemblies that form complex functional modules, and via convoluted biochemical pathways that themselves require a non-random spatial organization of components to proceed efficiently (Alberts, 1998, Aloy and Russell, 2004, Hartwell et al., 1999). Successful scientific exploration of cellular organization should therefore involve not only the identification and detailed characterization of parts, but also of their spatio-temporal interactions (Kohl et al., 2010). In order to understand the behaviour of an integrated system, such as a cell, we must consider how interacting networks (e.g. proteins, membrane structures, filaments, etc.) are arranged in 3D, how their arrangement enables and restricts their dynamics, and how these spatial relations are affected by both normal function (particularly in a contractile organ such as the heart) and pathological remodelling. Determination of *in situ* structure requires the development of cutting edge methods allowing for the cells to be preserved as close to their native state as possible, thus providing an accurate snapshot of the often unstable and transient molecular assemblies.

Many gaps in our current knowledge stem from the fact that structures we are studying are complex 3D objects, which are reduced to (at best - serial) 2D representations when analysed using conventional imaging approaches. Consequently, there is increasing appreciation and desire for detailed, high resolution, and – importantly – quantitative 3D analysis of structural and ultra-structural topology of biological samples. Unfortunately, many of the structures of interest are of dimensions equivalent to, or below, the diffraction limit of standard light microscopy, while at the same time being too complex to be easily studied by ultra-high resolution methods such as X-ray crystallography and nuclear magnetic resonance (NMR) spectroscopy.

Conventional transmission electron microscopy (TEM) offers an optimal X-Y resolution to study subcellular structures (1 nm in X-Y plane), yet it is inherently a 2D approach, with low maximum sample thickness (samples are typically 40–80 nm thick) leading to poor Z-coverage resolution. An advancement of this technique into the 3D domain came with the introduction of serial sectioning, allowing for pioneering insight into 3D cardiomyocyte ultrastructure (De Maziere et al., 1992, Johnson and Sommer, 1967). This approach is limited, however, in its Z-resolution, suffers from challenges in 3D integration of inherently deformed 2D sections, while also being very labour-intensive.

Over the last few years, scanning electron microscopy (SEM) has been used to overcome some of the limitations of conventional TEM. SEM uses an electron beam to visualise surface topology of a sample and is able to achieve resolution of 5 nm in X-Y (Sulkin et al., 2014, Glancy et al., 2015, Pinali and Kitmitto, 2014). When combined with serial block-face imaging (SBF-SEM), it is a method of choice for 3D nano-scale visualisation of biological structures over extended (for electron microscopy techniques) volumes, potentially linking observations from nano- to micro-scales (Denk and Horstmann, 2004, Heymann et al., 2009, Knott et al., 2008).

SBF-SEM development built on the use of an ultramicrotome mounted inside the vacuum chamber of a scanning electron microscope. Following imaging, a thin section of the freshly-scanned surface (usually 25 nm−100 nm) is cut from the block and discarded, before the ‘new’ block-face is imaged again (Peddie and Collinson, 2014). In FIB-SEM, instead of an ultramicrotome, an ion beam is used to burn-off, rather than mechanically cut, a thin layer (typically 5 nm–50 nm) of material from the sample surface (Knott et al., 2008, Peddie and Collinson, 2014). In both cases, one obtains stacks of inherently co-registered images that then can be aligned and reconstructed to reveal the 3D structure of interest within the spatial context of a cell or tissue (Villinger et al., 2012). Recently FIB-SEM has been successfully employed to visualize the nano-topography of muscle cells (Sulkin et al., 2014, Glancy et al., 2015, Pinali and Kitmitto, 2014), revealing the complex network of cardiac T-tubules (T-tub) as well as of mitochondrial “reticulum” both in cardiac and skeletal muscle cells. Technical challenges of the approach are associated with protecting the sample from degradation or warping by ion beam (heat damage) or blade interactions (mechanical damage). Additionally the device requires extreme levels of isolation from external sources of interference such as building vibrations, temperature fluctuations, ventilation drafts, etc. Furthermore, both SBF-SEM and FIB-SEM destroy the tissue sample as part of the observation process, and no re-imaging can be performed.

The introduction of electron tomography (ET) to biological research has allowed 3D reconstructions that are non-destructive in as far as the sample is concerned (Frank, 1992). The theory behind ET is very much like that of more well-known types of tomography (computed tomography or positron emission tomography) used routinely in clinical practice, with the differences that ET is based on electron beams (rather than X-rays or γ-rays), and that, in ET, the sample is rotated (rather than the beam source). In practice, ET involves sequential EM imaging, acquired at different angular orientations (usually between −70° and +70°) of a thick (typically 200 nm–300 nm) section. As for technical constrains, the prolonged electron beam exposure affects the plastic used for biological sample embedding, resulting in specimen deformation. This can be corrected for post-acquisition. Also, the overall volume covered is limited; however, tiling and stacking of serial high-resolution ET is possible, for example to reconstruct small cells (Hoog et al., 2007, Noske et al., 2008) or larger fragments of cardiomyocytes (up to 10–15 μm in X and/or Y).

The image stack obtained for ET is referred to as a tilt series. Post capture, tilt series are aligned and computationally merged by back-projecting each TEM image with appropriate weighing into a 3D tomogram of the imaged sample. A major determinant of resolution within the 3D volume is the accuracy of alignment of the tilt images. Inadequate image alignment will result in blurring or smearing of sample features. Additionally, exposure to the beam during the acquisition of a tilt series causes progressive geometric changes of the sample that need to be taken into account. A powerful method for ensuring consistent image alignment is based on the use of fiducial markers (typically uniformly-sized colloidal gold particles deposited on the specimen surface prior to data collection) as reference coordinates. Accordingly, post-acquisition image alignment is based on determination of the positions of these high-contrast regions in each image (Frank, 1992, Mastronarde, 2006, Ress et al., 1999, Kremer et al., 1996).

The final 3D tomogram exhibits resolution typically 40–100-fold superior to that achievable by confocal fluorescence imaging. It can be sliced virtually and viewed in any orientation. The resulting dimension of each computational slice is much thinner than one could physically cut for serial TEM, SBF-SEM, or FIB-SEM, leading to smaller image voxel dimensions (down to dimensions in the order of ∼1 nm^3^, compared to typically ∼125 nm^3^ or more with FIB-SEM). This represents a three orders of magnitude difference in spatial resolution of the 3D data (Mastronarde, 1997, McIntosh et al., 2005). However, there is an inherent limit to the range of tilt angles, due to the presence of the specimen holder, which results in a “missing wedge” of information in Fourier space. The primary effect of this missing wedge is a reduction in Z-resolution and anisotropic distortion (i.e. an elongation in Z-direction) (Frank, 2006). This problem has been addressed by the development of a dual-axis tomography, where the specimen is tilted around not one, but two mutually orthogonal axes. This produces two tilt series of the same sample, taking advantage of the non-destructive nature of tomographic imaging, and reduces the “missing wedge” to a much smaller “missing pyramid”. Each tilt series is computed separately into 3D tomograms, which are subsequently combined into one, yielding a data sat that exceeds both of the original tomograms in terms of fidelity and resolution (which is now more isotropic than either of the original tomograms) (Mastronarde, 1997, Penczek et al., 1995). A disadvantage of a two-axis method is the additional time required to obtain, align and calculate tomograms from two sets of images. The combining of the tomograms is also computationally demanding, although much of that process can be automated. Despite the extra effort, tilting around two axes is important for an accurate study of the 3D organization of complex biological structures. Dual-axis tomography is typically not worthwhile if the features studied are all in roughly the same orientation. However, when features of interest are running at a wide range of orientations within a specimen, tilting around a second axis overcomes the inherent limitations associated with single-axis tomography (Mastronarde, 1997).

In contrast to confocal light microscopy, where the 3D image is acquired via successive optical Z-slicing of the sample, in ET the slices are computationally extracted after the tomogram is reconstructed (the word “tomography” is derived from the Greek τόμος [*tómos*] meaning ‘a slice’ or ‘section’, and γραφὴ [*graphḗ*] meaning ‘a scripture’ or ‘a writing’). Slices can be computed in any orientation, and their minute minimum “thickness” allows one to visualise ultra-structural elements that might be hidden behind others (Kremer et al., 1996, Mastronarde, 2005, Subramaniam, 2005).

Thus, ET is an exciting method for studying complex cellular structures in 3D, although – like most EM-based approaches – it is extremely time-consuming and crucially depends on the development of suitable protocols for optimal sample preservation, imaging, and data analysis. Data are used for generation of 3D reconstructions of sub-structures; and this is referred to in the field as ‘modelling’. ET has been used to reconstruct whole cells (Hoog et al., 2007, Noske et al., 2008), with one of the largest tomographic volume of a whole cell that has been published to date being about 70 μm^3^ (*Saccharomyces pombe* cell, (Hoog et al., 2007)). An electron tomographic reconstruction of a whole cardiomyocyte (∼150 μm × 20 μm × 15 μm, i.e. 45,000 μm^3^ or more) would not be easily achieved, so FIB-SEM may be a more suitable approach for such a large-scale reconstruction.

An alternative is based on the notion that cardiomyocytes show axial periodicity (roughly every 2 μm) and rotational symmetry (with the assumption that a 45° wedge, from cell centre to periphery, should contain representative aspects of all sub-cellular structures). In this case, a meaningful reconstruction of cardiac myocytes may be possible from much reduced sub-volumes (e.g. [2 μm × 1/8 (20 μm × 15 μm], i.e. 75 μm^3^). This may need to be conducted once each at a cell end, one mid-cell region with, and one without nuclear presence. One could then reproduce a complete 3D map, at 1 nm^3^ voxel size, of an intact cardiomyocyte based on reconstructions of ‘only’ ∼250 μm^3^ of the cell, which – while challenging – is in the same ball-park as published whole-cell reconstructions.

A large body of evidence suggests that elaborate sub-cellular membrane structures play crucial roles in enabling cardiomyocyte function. Although the anatomy of specific ultra-structures (such as dyadic couplons) has been described using 2D EM of thin sections (Franzini-Armstrong et al., 1999, Sun et al., 1995), we lack accurate quantitative knowledge of many finer 3D structures of the cardiomyocyte and their spatial interrelation, with only a handful of studies attempting to tackle the issue thus far (Pinali and Kitmitto, 2014, Gherghiceanu and Popescu, 2011, Hayashi et al., 2009, Hong et al., 2014, Iribe et al., 2009, Leo-Macias et al., 2015, Pinali et al., 2013). These studies have used murine models, while non-murine (including rabbit) cardiac ultrastructure is largely unexplored at the nano-scale (with few exceptions (Pinali et al., 2013)). Given that rabbit cardiac structure and function resemble human heart better than murine heart cells (Jayasinghe et al., 2015, Bers, 2002) such investigations could provide valuable insights, in addition to allowing species comparisons.

In the following, we provide an overview of the utility of 3D ET in the identification, visualisation, and analysis of major sub-cellular structures including T-tub, different components of the sarcoplasmic reticulum (SR), microtubules (MT), and mitochondria (Mito), as well as their anatomical interrelations in rabbit ventricular cardiomyocytes.

## 2 Methods

All investigations reported in this manuscript were ethically approved and conformed to the UK Home Office guidance on the Operation of Animals (Scientific Procedures) Act of 1986.

### 2.1 Chemical fixation and imaging of native heart tissue

New Zealand white rabbit hearts (n = 6) were swiftly excised after euthanasia (pentobarbital injection), Langendorff-perfused with Krebs-Henseleit solution (containing [in mM]: NaCl 118, KCl 4.75, CaCl_2_ 2.5, NaHCO_3_ 24.8, MgSO_4_ 1.2, KH_2_PO_4_ 1.2, glucose 11, insulin 10 U/L; pH 7.4) and after, 5 min wash of the coronary circulation, cardioplegically arrested a using high-K^+^ (25 mM) version of Krebs-Henseleit solution. All solutions were controlled for iso-osmolality (295–305 mOsm; Knauer AG, Berlin). Cardioplegically arrested hearts were perfusion-fixed with iso-osmotic Karnovsky’s fixative (Karnovsky, 1965) (2.4% sodium cacodylate, 0.75% paraformaldehyde, 0.75% glutaraldehyde; 300 mOsm). Tissue fragments were excised from the left ventricle and washed with 0.1 M sodium cacodylate, post-fixed in 1% OsO_4_ for 1 h, dehydrated in graded acetone, and embedded in Epon-Araldite resin. Semi-thick (275 nm) sections were placed on formvar-coated slot-grids, post stained with 2% aqueous uranyl acetate and Reynold’s lead citrate. Colloidal gold particles (15 nm) were added to both surfaces of the sections to serve as fiducial markers for tilt series alignment.

Preparations were imaged at the Boulder Laboratory for 3D Electron Microscopy of Cells (University of Colorado, Boulder, CO) using an intermediate voltage electron microscope (Tecnai TF30; FEI, Eindhoven, The Netherlands) operating at 300 kV. Images were captured on a 4 K × 4 K charge-coupled device camera (UltraScan; Gatan, Pleasanton, CA) using the SerialEM software package (Ress et al., 1999). For imaging, the specimen holder was tilted from +60° to −60° at 1° intervals. For dual-axis tilt series the specimen was then rotated by 90° in the X-Y plane, and another +60° to −60° tilt series was taken. The images from each tilt-series were aligned by fiducial marker tracking and back-projected to generate two single full-thickness reconstructed volumes (tomograms), which were then combined to generate a single high-resolution 3D reconstruction of the original partial cell volume (Mastronarde, 1997). Isotropic voxel size was 1.206 nm. In some instances, tomograms were computed from montaged stacks, to increase the total reconstructed volume to up to 10 × 10 μm in X-Y. Biologically meaningful resolution was approximately 4 nm in X-Y. All tomograms were processed and analysed using IMOD software (Mastronarde, 2006), which was also used to generate 3D models of relevant structures of interest (Kremer et al., 1996). Models were smoothed and meshed to obtain the final 3D representation, in which spatial relations of various cardiomyocyte sub-structures were quantified.

## 3 Results

### 3.1 T-tubules

T-tub form a highly complex polymorphic network of membrane invaginations with diameters ranging from 20 to 450 nm, extending from the surface of the cell to its centre, often displaying complex branching and longitudinal extensions (Soeller and Cannell, 1999). The topology of the T-tub system of ventricular cardiomyocytes varies between species, from meshes in rat (Soeller and Cannell, 1999) to predominantly radial spokes in rabbit (Savio-Galimberti et al., 2008). Human T-tub architecture is thought to be intermediate between these two topologies, but closer to rabbit than rat or mouse (Jayasinghe et al., 2015). Available sub-micrometre scale data does not easily lend itself to exploration of details such as cross sectional aspect ratios (for assessment of T-tub surface to volume relation), let alone their dynamic modulation during the mechanical cycle of a cell (as attempted previously (McNary et al., 2012)). Additionally, no rabbit cardiomyocyte ET data have been published thus far, with studies focusing on rodent models.

**Fig. 1:**
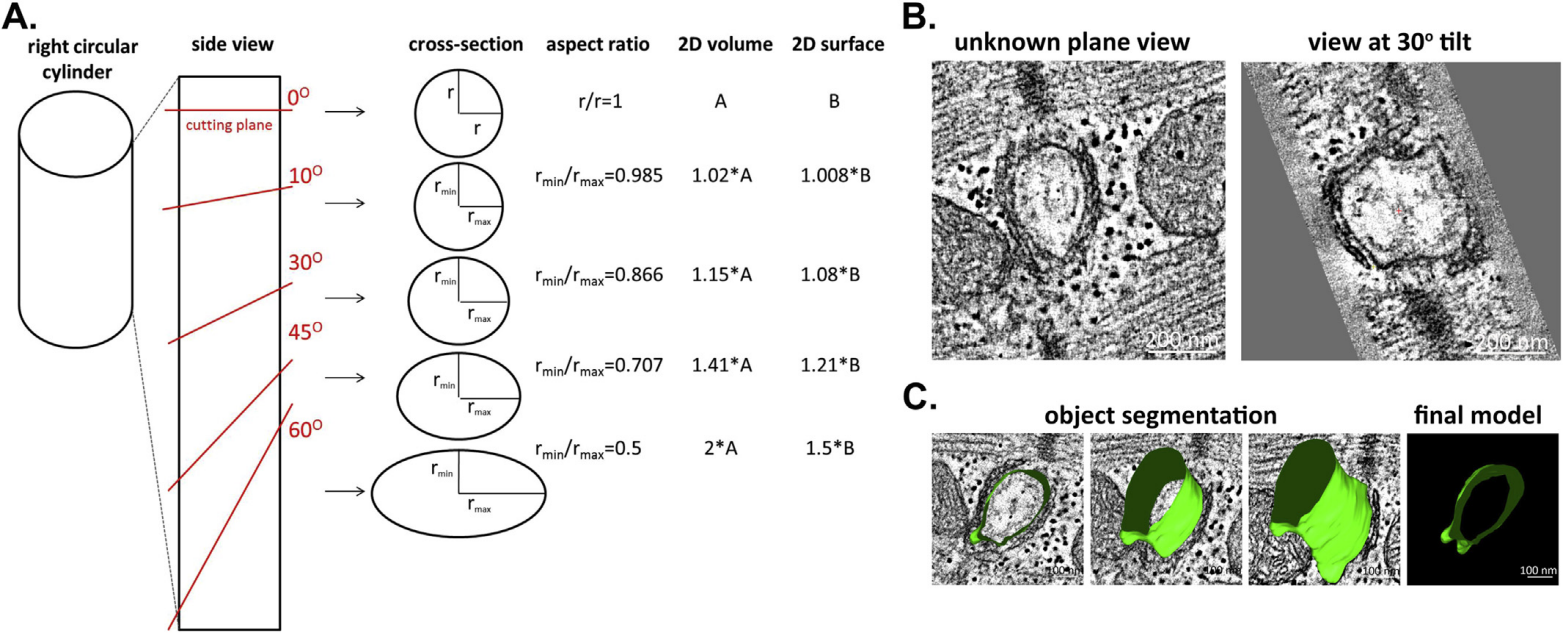
Electron tomography (ET) allows for accurate assessment of true T-tubule (T-tub) geometry. (A) Demonstration of the dependence of T-tub cross-section perception on the angle of the cutting plane. Lack of control over the cutting plane leads to potentially false estimation of T-tub shape, volume and surface area. Calculations were based on the law of sines. (B) Representative tomographic slices of the same T-tub at identical Z-height, showing the different perception of T-tub shape at different Y-plane tilts. Scale bar = 200 nm. (C) Example of manual T-tub segmentation along the Z-plane and a resulting 3D meshed model showing true T-tub geometry, with the final model (right) being an accurate image of T-tub cross-section perpendicularly to T-tub long axis. Segmentation was done using a tomographic montage (10 × 10 μm in X-Y). Scale bar = 100 nm.

**Fig. 2:**
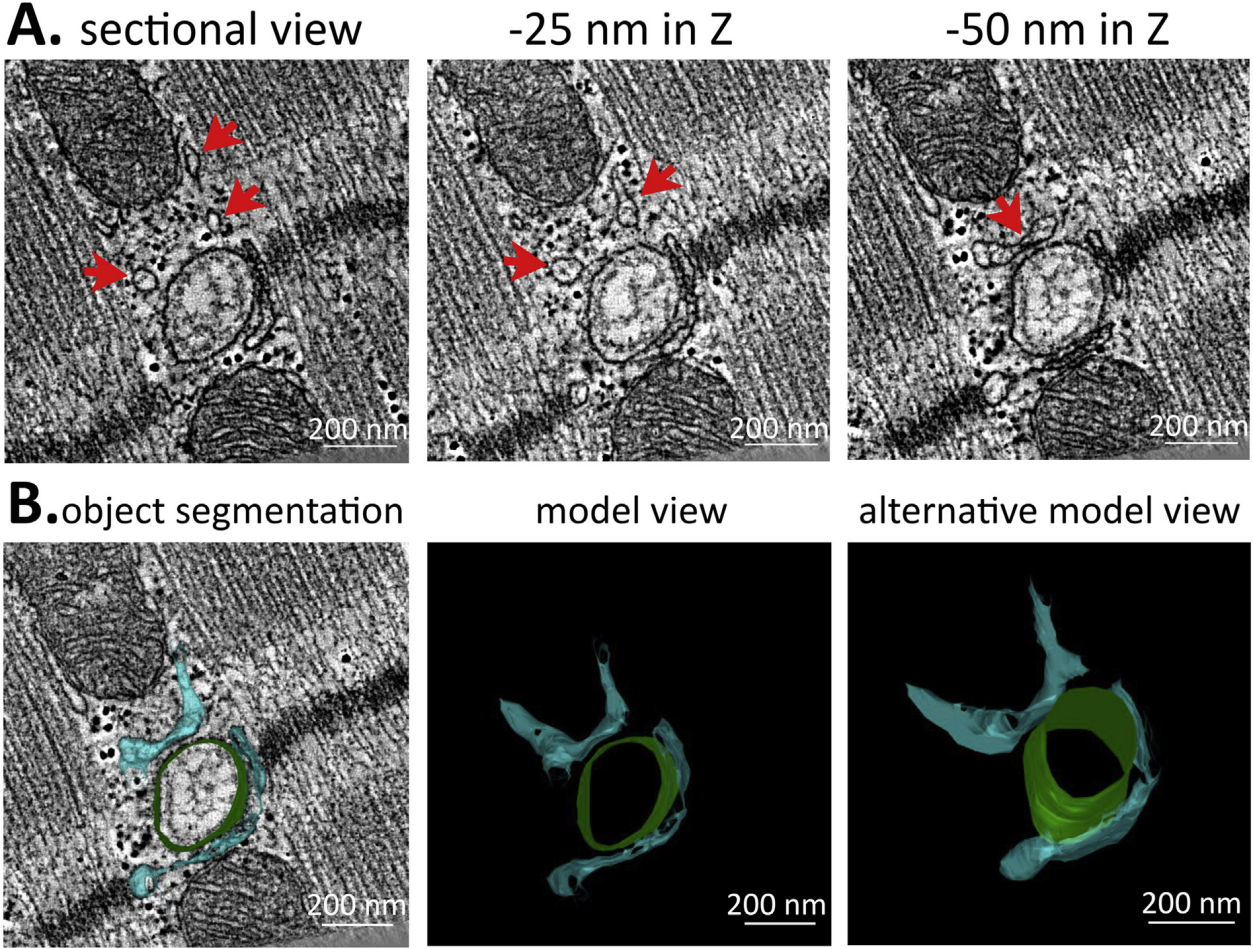
ET helps to properly identify elements of the junctional sarcoplasmic reticulum (jSR) network. (A) Three seemingly unconnected membranous structures (arrows) are demonstrated to be connected further down the Z-planes. Scale bar = 200 nm. (B) Example of jSR (blue) and T-tub (green) manual segmentation along the Z-plane and a resulting 3D meshed model showing close spatial relationship of the two structures. Scale bar = 200 nm.

Using ET it is possible to image, reconstruct and model cardiac T-tub in 3D. Here is where the major advantages of 3D ET over 2D TEM become apparent – namely, the ability to rotate the imaging plane, relative to the axis of T-tub or sarcomeric structures to reliably quantify parameters, such as T-tub cross section or sarcomere length (SL) in EM data. As any non-perpendicular cut through a cylinder would yield an ellipse (Fig. 1A,B), it is nearly impossible to assess with certainty aspects such as SL-dependent changes in the 3D shape of T-tub. ET supports determination and quantification the true geometry of the T-tub (Fig. 2B,C), including measurements of the absolute volume and surface area (in nm^3^ and nm^2^, respectively), as well as T-tub orientation relative to a reference structure of choice.

### 3.2 Sarcoplasmic reticulum

The SR is organized into at least two distinct functional (yet structurally interrelated) domains, the junctional SR (jSR) and the network SR (nSR), which together envelope the contractile apparatus (Franzini-Armstrong et al., 2005).

#### 3.2.1 Junctional SR

In cardiac muscle cells, the jSR is composed of planular cisternae wrapped around T-tub. The jSR network is extremely polymorphous, forming multiple discrete contacts both with transverse and longitudinal branches of T-tub system (Pinali and Kitmitto, 2014, Hayashi et al., 2009). Previous studies have found jSR to be wrapping around T-tub, and forming the dyadic clefts that are crucial for excitation-contraction coupling (Pinali and Kitmitto, 2014, Hayashi et al., 2009). SR then extends in parallel to sarcomeric fibres towards the next T-tub, forming a presumably continuous network. ET allows for proper identification of jSR elements which otherwise can appear as disconnected membrane vesicles in thin 2D sections (Fig. 2A). The Z-distance between the tomographic slices shown in Fig. 2A is 25 nm – i.e. three times below the thickness of serial thin sections used for EM data collection. It would not be possible to detect or predict structural changes, occurring over such distances, using conventional EM. In addition, following the jSR throughout the ET data volume enables one to construct realistic jSR-T-tub models (Fig 2B), which are needed for quantification of volume, surface area, interaction with other sub-cellular structures, as well as for characterization and computational modelling of the dyadic cleft (as previously attempted in (Greenstein and Winslow, 2002)).

#### 3.2.2 Network SR

**Fig. 3:**
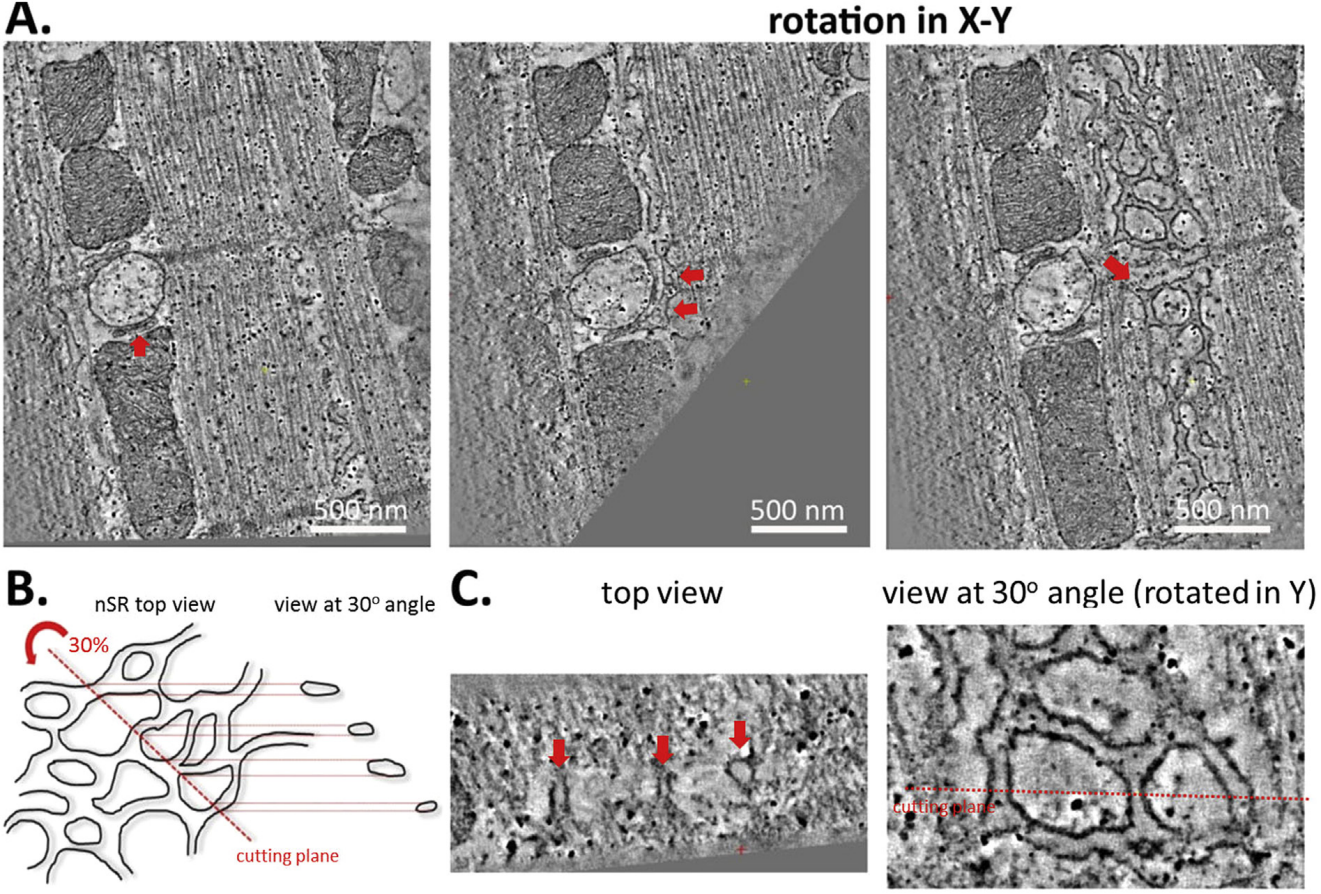
ET allows the network SR (nSR) to be distinguished and followed throughout the cell. (A) jSR (left panel, arrow) and nSR can be accurately followed by 3D image rotation (middle and right, arrows pointing out the continuity between jSR and nSR). Scale bar = 500 nm. (B) A schematic representation of difficulties in identifying nSR in 2D micrographs if the section is cut in a sub-optimal plane. (C) An example of nSR network visualised at a different virtual “cutting plane” – what appears to be unidentifiable vesicles (right, arrows) is in fact part of larger nSR web.

In cardiomyocytes, sarcomeric proteins are surrounded by nSR, a loose web of flattened tubules that resemble smooth ER in other cell types (Pinali and Kitmitto, 2014, Ackermann et al., 2011, Franzini-Armstrong, 1999). Due to its structural complexity, nSR is notoriously difficult to identify accurately in 2D EM images. ET allows for full appreciation of the complex structure of nSR and enables one to follow its intricate branching throughout the cell (Fig. 3A). It also allows the observer to identify membrane fragments, seemingly floating in space, as parts of a larger nSR network (Fig. 3B). By 3D analysis one can locate points of contact of nSR and jSR, and obtain quantitative characteristics that are currently still scarce, for example for inclusion in computational modelling of cardiomyocyte Ca^2+^ dynamics (Hake et al., 2012).

### 3.3 Microtubules

**Fig. 4:**
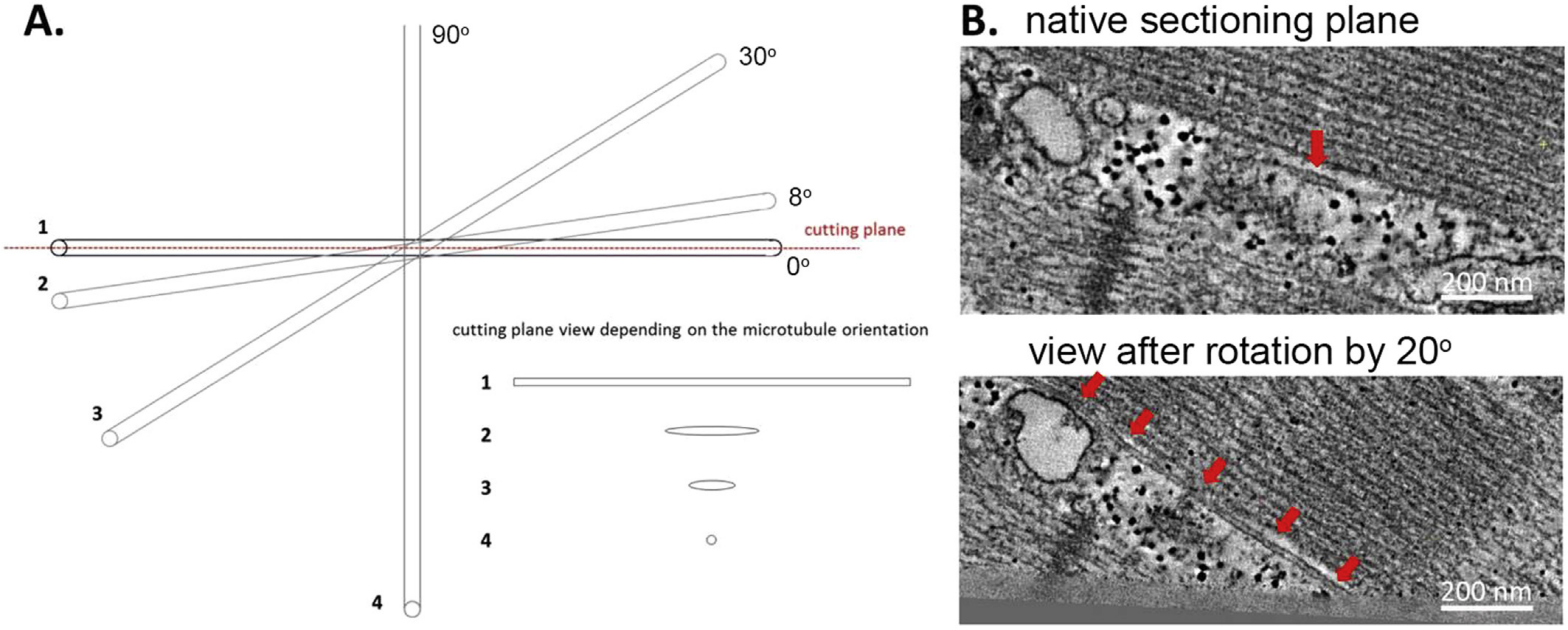
Proper identification of microtubules (MT) in TEM data is hindered by their often deceptive appearance that depends on their orientation relative to the cutting plane. (A) A schematic representation of the appearance of a microtubule in 2D section depending on its orientation relative to the cutting plane. (B) MT that may be missed in the native cutting plane (top) can be identified (arrows) and followed in 3D tomograms using ‘image rotation tools. Scale bar = 200 nm.

MT are filamentous, highly dynamic structures, undergoing constant assembly/disassembly that is dependent on a range of factors, including the mechanical state of the cell (Goldstein and Entman, 1979). ET has previously been used to study MT, and has proven to be an invaluable tool in both identifying and analysing these labile structures (Iribe et al., 2009, Nannas et al., 2014). The filamentous shape of microtubules (with diameters of 20–25 nm, yet lengths reaching up to 50 μm) makes them difficult to recognize in TEM, where – unless the mechanical cutting plane happens to match that of the MT – they appear as enigmatic round objects with various cross sections (Fig. 4A and B). Because a tomographic slice is a sample through a 3D volume, it can be rotated and viewed in any orientation. Thus, ET can help in the identification and tracking of MT throughout the cell, even if they change orientation (Fig. 4B), as well as in deciphering the spatial interrelations of these structures with other intracellular complexes, such as SR or T-tub, and their functional relevance (e.g. for mechanical modulation of Ca^2+^ spark activity) (Iribe et al., 2009).

Without ET, the chance of cutting the section in parallel and in the plane of a MT is small. In a 2D section MT will appear as an ellipse if considering a microtubule as a simple cylinder and excepting the case when the slice is aligned perfectly with, or perpendicular to, the microtubule as seen in Fig. 4A. No matter what orientation the cylinder is cut at, the minor diameter of the ellipse will equal the true diameter (d) of the microtubule. The major diameter of the ellipse however will depend on the angle of cutting plane relative to the MT long axis, as seen with T-tub in Fig. 1A. Specifying a length (l) (or ratio of length to diameter) required to recognize a microtubule allows one to identify the cutting angle at which identification can occur. The rotation of the microtubule within the XY-plane does not matter for identification purposes, thus the probability of the major diameter being lower than the required length can be found by dividing the cutting angle by 90°. The probability (p) of correctly identifying a microtubule is thus:

$$p=\;\cos^{-1}\left(\frac{l}{d}\right)$$

Fot MT with a length of 50 μm and a diameter of 20 nm, the probability of seeing the full MT when sectioning is 0.03%. However, correctly identifying a sub-cellular structure as a MT does not necessarily require visualising its full length. If one assumes that seeing a structure of the appropriate dimensions where the length is at least five-fold greater than the diameter is sufficient to identify a MT then the probability is 12.8%. Comparing these probabilities with the near 100% identifiability of microtubules in an ET data set further demonstrates its utility.

### 3.4 Mitochondria

**Fig. 5:**
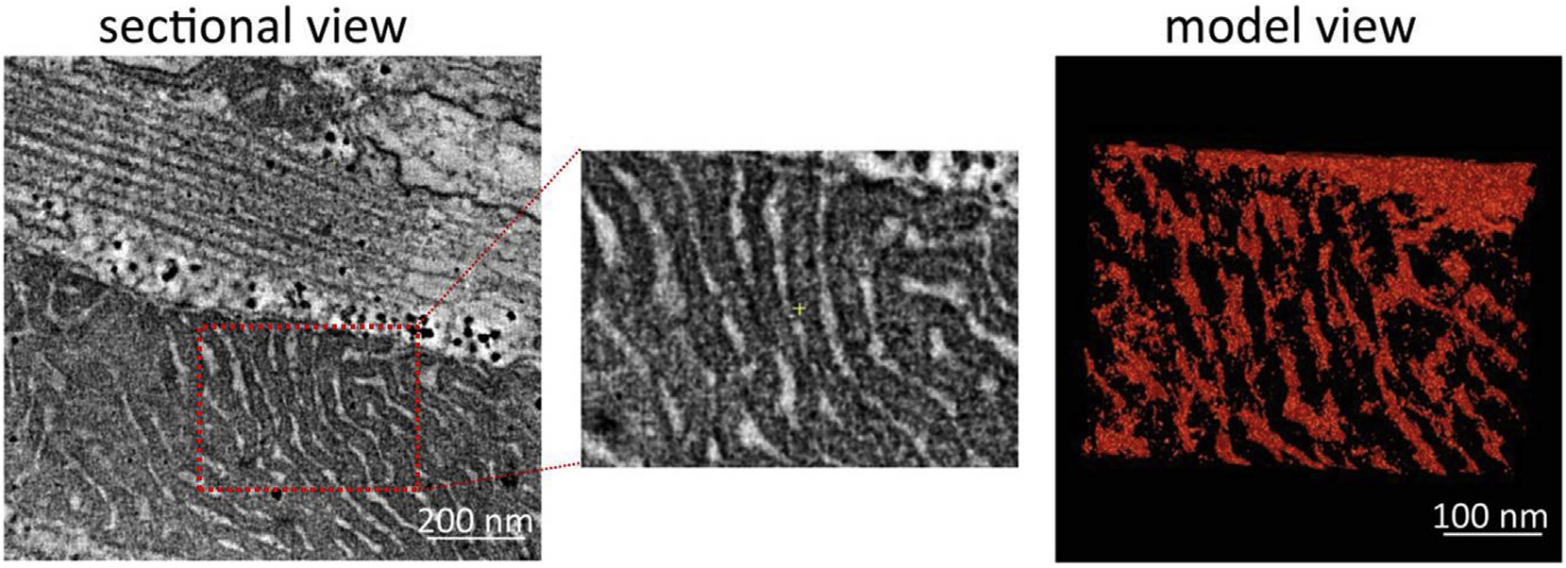
Mitochondrial (Mito) structure can be modelled in 3D using ET. Right - an example 3D model (built semi-automatically using Isosurface function of IMOD software, generating a surface at places where the intensity values cross a threshold value set by user) of a Mito segment (left, and middle – magnified view), with detailed representation of cristae demonstrating their intricate convolutedness. Scale bar = 200 nm (left) and 100 nm (right).

Mito network dynamics is a very recent topic of research in the field of cardiac sciences (Aon et al., 2006), with a focus on the mechanisms involved in functional integration, morphological changes, and mobility of Mito (Kanzaki et al., 2010, Piquereau et al., 2013) and their effect on energy production, ion homeostasis, free radical production and apoptosis. *Cristae* are folds of the Mito inner membrane that provide an increase in the surface area (Mannella, 2006), as well as being involved in other processes occurring outside Mito (Kanzaki et al., 2010, Piquereau et al., 2013). The tortuous structure of these inner membrane folds does not lend itself to exploration; however ET allows tracing and modelling of the cristae in 3D (Fig. 5), and thus detailed assessment of Mito morphology and remodelling (Kalkhoran et al., 2014).

## 4 Discussion

The 3D topologies of rabbit cardiomyocyte intracellular organelles can be mapped using ET. We present high-resolution ultrastructural data (for convenience as tomographic slices) and illustrate methods of structural rendering of crucial elements for cardiac function, including T-tub, SR, MT and Mito.

The densely packed intracellular structure of cardiomyocytes does not lend itself to reconstruction from 2D section data. ET gives an opportunity to characterise elaborate 3D sub-cellular membrane and filamentous structures. Structures of interest can be followed and traced, allowing for examination of features that otherwise might escape notice (Frey et al., 2006). For example, MT may escape detection, while a tubular membraneous structure such as T-tub may have a perfectly round cross-section but will appear elliptical when cut at an angle. In addition, one cannot accurately trace and confirm physical connections between structural components observed in 2D sections, or quantify their size and interrelation. Last, but not least, ET allows user-defined display and exploration, as structures of interest can be rotated, panned, and zoomed. This is aided by 3D models, generated by segmentation of 3D data sets, which allow extraction of quantitative data (e.g. volume, surface area) and aid development of more realistic computational cell models. This information can be used to improve existing structural and functional models of excitation-contraction coupling (e.g. of spatio-temporal Ca^2+^ dynamics) (Hake et al., 2012, Fernandez, 2012, Kekenes-Huskey et al., 2012, Rajagopal et al., 2015).

The quality and reliability of 3D reconstruction depends crucially on sample quality, image acquisition, alignment accuracy, angular sampling range, spatial coverage, and reconstruction algorithms. The field of ET is advancing rapidly, with improvements in all named critical areas. Sample vitrification is one such recent development, which improves the preservation of molecular structures (Lučić et al., 2013, Hsieh et al., 2006, Hurbain and Sachse, 2011). Classic methods of sample fixation using room temperature, aldehyde-based fixation and dehydration, even if isosmotic (or using ‘lightly fixed’ preparations), bear the risk of altering the structure of biological samples, leading to fixation artefacts and subsequent misconceptions about cellular organization (Heuser, 2002). By imaging hydrated samples in EM one avoids dehydration-induced structural alterations such as flattening and collapse of intracellular membranous structures (Gruska et al., 2008). Vitrification can be achieved either following plunge-freezing (Dubochet et al., 1988) or high-pressure freezing (Studer et al., 2008). High-pressure frozen samples may be dehydrated by freeze substitution (while remaining at temperatures below the vitirification temperature of water), embedded in resin, sectioned, and then imaged at room temperature (Hurbain and Sachse, 2011), or imaged in the native and frozen state by cryo-ET (Lučić et al., 2013, Hsieh et al., 2006, Tocheva et al., 2010).

Along with sample preparation, another determinant of final tomogram quality is sample thickness. An electron beam has a penetration depth of about 200 nm for an accelerating voltage of 120 kV and about 500 nm for 300 kV. As the trans-section path length doubles at 60° tilt, and nearly triples at 70°, this restricts useful sample thickness values to 100–300 nm, depending on the acceleration voltages used. Larger sub-volumes of a cell can be reconstructed with serial section ET approaches, where individual ET data sets are tiled together in-plane, or stacked across the z-direction. The constant improvement of cameras, as well as the introduction of direct detection devices (as opposed to charge-coupled devices that are currently widespread) allows for better detection of electrons and avoidance of blurring and distortion due to the generation and transfer of photons (Milazzo et al., 2011).

Thus, ET is an exciting, rapidly improving addition to the repertoire of tools aimed at exploring nano to meso scale (from 10^−9^ to 10^−6^ m) structure of cardiac cells. Electron-based 2D imaging played a key role in establishing structural cell biology as a discipline, by producing fundamental insights into cellular organization. Development of new sample preparation methods, imaging modalities and unbiased analysis tools for 3D ultrastructural reconstruction means that electron-based imaging remains at the forefront of progress in biophysics and cell biology, including the cardiovascular sciences.

## Editors' note

Please see also related communications in this issue by Quinn and Kohl (2016) and Gemmel et al. (2016).

## References

[bib1] Ackermann M.A., Ziman A.P., Strong J., Zhang Y., Hartford A.K., Ward C.W. (2011). Integrity of the network sarcoplasmic reticulum in skeletal muscle requires small ankyrin 1. J. Cell Sci..

[bib2] Alberts B. (1998). The cell as a collection of protein machines: preparing the next generation of molecular biologists. Cell.

[bib3] Aloy P., Russell R.B. (2004). Ten thousand interactions for the molecular biologist. Nat. Biotechnol..

[bib4] Aon M.A., Cortassa S., O’Rourke B. (2006). The fundamental organization of cardiac mitochondria as a network of coupled oscillators. Biophys. J..

[bib5] Asano S., Engel B.D., Baumeister W. (2016 Jan 29). In situ cryo-electron tomography: a post-reductionist approach to structural biology. J. Mol. Biol..

[bib6] Bers D.M. (2002). Cardiac excitation-contraction coupling. Nature.

[bib7] De Maziere A.M., van Ginneken A.C., Wilders R., Jongsma H.J., Bouman L.N. (1992). Spatial and functional relationship between myocytes and fibroblasts in the rabbit sinoatrial node. J. Mol. Cell Cardiol..

[bib8] Denk W., Horstmann H. (2004). Serial block-face scanning electron microscopy to reconstruct three-dimensional tissue nanostructure. PLoS Biol..

[bib9] Dubochet J., Adrian M., Chang J.J., Homo J.C., Lepault J., McDowall A.W. (1988). Cryo-electron microscopy of vitrified specimens. Q. Rev. Biophys..

[bib10] Fernandez J.J. (2012). Computational methods for electron tomography. Micron (Oxf. Engl. 1993).

[bib11] Frank J. (1992). Electron Tomography: Three-dimensional Imaging with the Transmission Electron Microscope (Mathematical Concepts and Methods in Science and Engineering).

[bib12] Frank J. (2006). Electron Tomography. Methods for Three-dimensional Visualization of Structures in the Cell.

[bib13] Franzini-Armstrong C. (1999). The sarcoplasmic reticulum and the control of muscle contraction. FASEB J..

[bib14] Franzini-Armstrong C., Protasi F., Ramesh V. (1999). Shape, size, and distribution of Ca(2+) release units and couplons in skeletal and cardiac muscles. Biophys. J..

[bib15] Franzini-Armstrong C., Protasi F., Tijskens P. (2005). The assembly of calcium release units in cardiac muscle. Ann. N. Y. Acad. Sci..

[bib16] Frey T.G., Perkins G.A., Ellisman M.H. (2006). Electron tomography of membrane-bound cellular organelles. Annu. Rev. Biophys. Biomol. Struct..

[bib68] Gemmell P., Burrage K., Rodriguez B., Quinn T.A. (2016). 2016. Rabbit-specific computational modelling of ventricular cell electrophysiology: using population models to explore variability to ischemia. Prog Biophys Mol Biol.

[bib17] Gherghiceanu M., Popescu L.M. (2011). Heterocellular communication in the heart: electron tomography of telocyte-myocyte junctions. J. Cell. Mol. Med..

[bib18] Glancy B., Hartnell L.M., Malide D., Yu Z.-X., Combs C.A., Connelly P.S. (2015). Mitochondrial reticulum for cellular energy distribution in muscle. Nature.

[bib19] Goldstein M.A., Entman M.L. (1979). Microtubules in mammalian heart muscle. J. Cell Biol..

[bib20] Greenstein J.L., Winslow R.L. (2002). An integrative model of the cardiac ventricular myocyte incorporating local control of Ca2+ release. Biophys. J..

[bib21] Gruska M., Medalia O., Baumeister W., Leis A. (2008). Electron tomography of vitreous sections from cultured mammalian cells. J. Struct. Biol..

[bib22] Hake J., Edwards A.G., Yu Z., Kekenes-Huskey P.M., Michailova A.P., McCammon J.A. (2012). Modelling cardiac calcium sparks in a three-dimensional reconstruction of a calcium release unit. J. Physiol..

[bib23] Hartwell L.H., Hopfield J.J., Leibler S., Murray A.W. (1999). From molecular to modular cell biology. Nature.

[bib24] Hayashi T., Martone M.E., Yu Z., Thor A., Doi M., Holst M.J. (2009). Three-dimensional electron microscopy reveals new details of membrane systems for Ca2+ signaling in the heart. J. Cell Sci..

[bib25] Heuser J. (2002). Whatever happened to the ’microtrabecular concept’?. Biol. Cell Auspices Eur. Cell Biol. Organ..

[bib26] Heymann J.A.W., Shi D., Kim S., Bliss D., Milne J.L.S., Subramaniam S. (2009). 3D Imaging of mammalian cells with ion-abrasion scanning electron microscopy. J. Struct. Biol..

[bib27] Hong T., Yang H., Zhang S.-S., Cho H.C., Kalashnikova M., Sun B. (2014). Cardiac BIN1 folds T-tubule membrane, controlling ion flux and limiting arrhythmia. Nat. Med..

[bib28] Hoog J.L., Schwartz C., Noon A.T., O’Toole E.T., Mastronarde D.N., McIntosh J.R. (2007). Organization of interphase microtubules in fission yeast analyzed by electron tomography. Dev. Cell.

[bib29] Hsieh C.E., Leith A., Mannella C.A., Frank J., Marko M. (2006). Towards high-resolution three-dimensional imaging of native mammalian tissue: electron tomography of frozen-hydrated rat liver sections. J. Struct. Biol..

[bib30] Hurbain I., Sachse M. (2011). The future is cold: cryo-preparation methods for transmission electron microscopy of cells. Biol. Cell Auspices Eur. Cell Biol. Organ..

[bib31] Iribe G., Ward C.W., Camelliti P., Bollensdorff C., Mason F., Burton R.A. (2009). Axial stretch of rat single ventricular cardiomyocytes causes an acute and transient increase in Ca2+ spark rate. Circ. Res..

[bib32] Jayasinghe I.D., Clowsley A.H., Munro M., Hou Y., Crossman D.J., Soeller C. (2015). Revealing T-tubules in striated muscle with new optical super-resolution microscopy techniquess. Eur. J. Transl. Myol..

[bib33] Johnson E.A., Sommer J.R. (1967). A strand of cardiac muscle. Its ultrastructure and the electrophysiological implications of its geometry. J. Cell Biol..

[bib34] Kalkhoran S., Hall A., Cole A., White I., Yellon D., Hausenloy D. (2014). 3D electron microscopy tomography to assess mitochondrial morphology in the adult heart. Heart.

[bib35] Kanzaki Y., Terasaki F., Okabe M., Otsuka K., Katashima T., Fujita S. (2010). Giant mitochondria in the myocardium of a patient with mitochondrial cardiomyopathy: transmission and 3-Dimensional scanning electron microscopy. Circulation.

[bib36] Karnovsky M.J. (1965). A formaldehyde-glutaraldehyde fixative of high osmolarity for use in electron microscopy. J. Cell Biol..

[bib37] Kekenes-Huskey P.M., Cheng Y., Hake J.E., Sachse F.B., Bridge J.H., Holst M.J. (2012). Modeling effects of L-type ca(2+) current and na(+)-ca(2+) exchanger on ca(2+) trigger flux in rabbit myocytes with realistic T-tubule geometries. Front. Physiol..

[bib38] Knott G., Marchman H., Wall D., Lich B. (2008). Serial section scanning electron microscopy of adult brain tissue using focused ion beam milling. J. Neurosci..

[bib39] Kohl P., Crampin E.J., Quinn T.A., Noble D. (2010). Systems biology: an approach. Clin. Pharmacol. Ther..

[bib40] Kremer J.R., Mastronarde D.N., McIntosh J.R. (1996). Computer visualization of three-dimensional image data using IMOD. J. Struct. Biol..

[bib41] Leo-Macias A., Liang F.X., Delmar M. (2015). Ultrastructure of the intercellular space in adult murine ventricle revealed by quantitative tomographic electron microscopy. Cardiovasc. Res..

[bib42] Lučić V., Rigort A., Baumeister W. (2013). Cryo-electron tomography: the challenge of doing structural biology in situ. J. Cell Biol..

[bib43] Mannella C.A. (2006). Structure and dynamics of the mitochondrial inner membrane cristae. Biochim. Biophys. Acta (BBA) Mol. Cell Res..

[bib44] Mastronarde D.N. (1997). Dual-axis tomography: an approach with alignment methods that preserve resolution. J. Struct. Biol..

[bib45] Mastronarde D.N. (2005). Automated electron microscope tomography using robust prediction of specimen movements. J. Struct. Biol..

[bib46] Mastronarde D. (2006). Tomographic reconstruction with the IMOD software package. Microsc. Microanal..

[bib47] McIntosh R., Nicastro D., Mastronarde D. (2005). New views of cells in 3D: an introduction to electron tomography. Trends Cell Biol..

[bib48] McNary T.G., Spitzer K.W., Holloway H., Bridge J.H., Kohl P., Sachse F.B. (2012). Mechanical modulation of the transverse tubular system of ventricular cardiomyocytes. Prog. Biophys. Mol. Biol..

[bib49] Milazzo A.C., Cheng A., Moeller A., Lyumkis D., Jacovetty E., Polukas J. (2011). Initial evaluation of a direct detection device detector for single particle cryo-electron microscopy. J. Struct. Biol..

[bib50] Nannas N.J., O’Toole E.T., Winey M., Murray A.W. (2014). Chromosomal attachments set length and microtubule number in the Saccharomyces cerevisiae mitotic spindle. Mol. Biol. Cell.

[bib51] Noske A.B., Costin A.J., Morgan G.P., Marsh B.J. (2008). Expedited approaches to whole cell electron tomography and organelle mark-up in situ in high-pressure frozen pancreatic islets. J. Struct. Biol..

[bib52] Peddie C.J., Collinson L.M. (2014). Exploring the third dimension: volume electron microscopy comes of age. Micron (Oxf. Engl. 1993).

[bib53] Penczek P., Marko M., Buttle K., Frank J. (1995). Double-tilt electron tomography. Ultramicroscopy.

[bib54] Pinali C., Kitmitto A. (2014). Serial block face scanning electron microscopy for the study of cardiac muscle ultrastructure at nanoscale resolutions. J. Mol. Cell. Cardiol..

[bib55] Pinali C., Bennett H., Davenport J.B., Trafford A.W., Kitmitto A. (2013). Three-dimensional reconstruction of cardiac sarcoplasmic reticulum reveals a continuous network linking transverse-tubules: this organization is perturbed in heart failure. Circ. Res..

[bib56] Piquereau J., Caffin F., Novotova M., Lemaire C., Veksler V., Garnier A. (2013). Mitochondrial dynamics in the adult cardiomyocytes: which roles for a highly specialized cell?. Front. Physiol..

[bib57] Rajagopal V., Bass G., Walker C.G., Crossman D.J., Petzer A., Hickey A. (2015). Examination of the effects of heterogeneous organization of RyR clusters, myofibrils and mitochondria on Ca2+ release patterns in cardiomyocytes. PLoS Comput. Biol..

[bib67] Quinn T.A., Kohl P. (2016). 2016. Rabbit models of cardiac mechano-electric and mechano-mechanical coupling. Prog Biophys Mol Biol.

[bib58] Ress D., Harlow M.L., Schwarz M., Marshall R.M., McMahan U.J. (1999). Automatic acquisition of fiducial markers and alignment of images in tilt series for electron tomography. J. Electron. Microsc..

[bib59] Savio-Galimberti E., Frank J., Inoue M., Goldhaber J.I., Cannell M.B., Bridge J.H. (2008). Novel features of the rabbit transverse tubular system revealed by quantitative analysis of three-dimensional reconstructions from confocal images. Biophys. J..

[bib60] Soeller C., Cannell M.B. (1999). Examination of the transverse tubular system in living cardiac rat myocytes by 2-photon microscopy and digital image-processing techniques. Circ. Res..

[bib61] Studer D., Humbel B.M., Chiquet M. (2008). Electron microscopy of high pressure frozen samples: bridging the gap between cellular ultrastructure and atomic resolution. Histochem. Cell Biol..

[bib62] Subramaniam S. (2005). Bridging the imaging gap: visualizing subcellular architecture with electron tomography. Curr. Opin. Microbiol..

[bib63] Sulkin M.S., Yang F., Holzem K.M., Van Leer B., Bugge C., Laughner J.I. (2014). Nanoscale three-dimensional imaging of the human myocyte. J. Struct. Biol..

[bib64] Sun X.H., Protasi F., Takahashi M., Takeshima H., Ferguson D.G., Franzini-Armstrong C. (1995). Molecular architecture of membranes involved in excitation-contraction coupling of cardiac muscle. J. Cell Biol..

[bib65] Tocheva E.I., Li Z., Jensen G.J. (2010). Electron cryotomography. Cold Spring Harbor Perspect. in Biol..

[bib66] Villinger C., Gregorius H., Kranz C., Hohn K., Munzberg C., von Wichert G. (2012). FIB/SEM tomography with TEM-like resolution for 3D imaging of high-pressure frozen cells. Histochem. Cell Biol..

